# Diagnostic Value of the Vertebral Right Heart Index (VRHi) for Identifying Pulmonary Hypertension in Dogs with Heartworm Disease

**DOI:** 10.3390/ani16203239

**Published:** 2026-10-15

**Authors:** Jorge Isidoro Matos, Claudia Díaz-Rocio, Sara Nieves García-Rodríguez, Alicia Caro-Vadillo, Laín García-Guasch, Eva Mohr-Peraza, José Alberto Montoya-Alonso, Elena Carreton

**Affiliations:** 1 Internal Medicine, Faculty of Veterinary Medicine, Research Institute of Biomedical and Health Sciences (IUIBS), University of Las Palmas de Gran Canaria, 35016 Las Palmas de Gran Canaria, Spain; jorge.matos@ulpgc.es (J.I.M.); claudia.diaz106@alu.ulpgc.es (C.D.-R.); aliciac@vet.ucm.es (A.C.-V.); lain.garcia@ivcevidensia.es (L.G.-G.); eva.mohr@ulpgc.es (E.M.-P.); alberto.montoya@ulpgc.es (J.A.M.-A.); elena.carreton@ulpgc.es (E.C.); 2 Hospital Clínico Veterinario, Faculty of Veterinary Medicine, Universidad Complutense de Madrid (UCM), 28040 Madrid, Spain; 3 Hospital Veterinari Molins, IVC Evidensia, 08750 Barcelona, Spain; 4 Hospital Veterinaria del Mar, IVC Evidensia, 08005 Barcelona, Spain

**Keywords:** *Dirofilaria immitis*, heartworm disease, echocardiography, pulmonary hypertension, right vertebral heart index, thoracic radiography, vertebral heart size, veterinary imaging

## Abstract

Pulmonary hypertension (PH) is a common complication of canine heartworm disease caused by *Dirofilaria immitis* and is associated with right-sided cardiac remodelling. This study evaluated the diagnostic value of the Right Vertebral Heart Index (VRHi) for identifying dogs with a high echocardiographic probability of PH and its association with echocardiographic indices of right-sided cardiac remodelling. Fifty-four dogs from Gran Canaria were included: 20 healthy controls and 34 dogs naturally infected with *D. immitis*, classified according to the presence or absence of a high echocardiographic probability of PH. Thoracic radiographs were obtained without sedation, and the vertebral heart size (VHS) and VRHi were measured using DICOM software. Heartworm infection was confirmed using a commercial antigen detection test. Echocardiography was performed to assess parasite burden, determine the probability of PH, and evaluate right-sided cardiac remodelling. Dogs with a high echocardiographic probability of PH showed more frequent clinical signs and greater echocardiographic evidence of right-sided cardiac remodelling than dogs without PH. The VRHi values differed significantly among the three study groups and were highest in dogs with a high echocardiographic probability of PH. The VRHi was significantly correlated with echocardiographic indicators of right-sided remodelling. The right lateral projection showed the highest AUC among VRHi measurements (AUC = 0.835), although its AUC did not differ significantly from that of conventional VHS (AUC = 0.764; DeLong *p* = 0.519). These findings suggest that VRHi may provide a simple and objective complementary radiographic parameter in dogs with heartworm disease and suspected PH.

## Simple Summary

Pulmonary hypertension is a serious complication of canine heartworm disease caused by *Dirofilaria immitis* and is associated with right-sided cardiac remodelling and a poorer prognosis. Although echocardiography is the reference method for diagnosing pulmonary hypertension, it is not always readily available in clinical practice. Thoracic radiography is more widely accessible, but conventional radiographic measurements have limited ability to evaluate right-sided cardiac remodelling. This study assessed the diagnostic value of the Right Vertebral Heart Index (VRHi), a radiographic measurement specifically designed to estimate right heart size, in dogs naturally infected with *Dirofilaria immitis*. Dogs with high echocardiographic probability of pulmonary hypertension had significantly higher VRHi values than healthy dogs and infected dogs without pulmonary hypertension, and these measurements were associated with echocardiographic indicators of right-sided cardiac remodelling. In addition, VRHi showed good diagnostic performance for identifying dogs with a high echocardiographic probability of PH. These findings suggest that VRHi may be a practical and objective complementary tool for the radiographic assessment of right-sided cardiac remodelling and pulmonary hypertension in dogs with heartworm disease.

## 1. Introduction

*Dirofilaria immitis* is a mosquito-borne nematode that causes cardiopulmonary dirofilariosis in dogs and other carnivores and is also responsible for human pulmonary dirofilariosis [1,2]. Adult worms reside in the pulmonary arteries, where they induce endothelial injury, vascular remodelling, thrombosis, and progressive increases in pulmonary vascular resistance, ultimately leading to pulmonary hypertension (PH) [3]. According to the American College of Veterinary Internal Medicine (ACVIM) consensus statement, heartworm disease is classified as a cause of precapillary PH (group 5) [4]. Persistent elevation of pulmonary arterial pressure increases right ventricular afterload, resulting in right-sided cardiac remodelling and, in advanced cases, right-sided congestive heart failure [4]. Approximately 40% of naturally infected dogs develop PH, making it one of the most clinically relevant complications of heartworm disease [5,6].

Although right heart catheterization remains the reference standard for measuring pulmonary arterial pressure, its invasive nature limits its use in veterinary medicine. Consequently, Doppler echocardiography is currently the preferred non-invasive method for estimating the probability of PH based on tricuspid regurgitation velocity and additional echocardiographic findings [4]. Nevertheless, echocardiography is not always readily available and requires specialized equipment and expertise. Thoracic radiography therefore remains an essential complementary imaging modality because it is inexpensive, widely accessible, and provides valuable information on both pulmonary vascular changes and cardiac remodelling [7,8].

Radiographic abnormalities associated with PH include enlargement of the pulmonary trunk and pulmonary arteries, peripheral arterial tortuosity and pruning, right ventricular enlargement, and signs of right-sided heart failure, such as caudal vena cava distension and pleural or abdominal effusion [9,10,11]. However, most of these findings are subjective and often become evident only in advanced disease, highlighting the need for objective radiographic measurements [12,13].

Most quantitative radiographic indices currently used in veterinary cardiology, including the vertebral heart size (VHS), vertebral left atrial size (VLAS), radiographic left atrial dimension (RLAD), and thoracic inlet heart size (TIHS), were developed to evaluate left-sided cardiac remodelling and provide limited information on right-sided remodelling or PH [14,15,16,17]. Among the few objective radiographic methods specifically proposed for the assessment of PH are the ratios between the right cranial pulmonary artery and the fourth rib (CrPA/R4) and between the right caudal pulmonary artery and the ninth rib (CdPA/R9), which have shown diagnostic value in dogs with heartworm disease [5].

More recently, the Right Vertebral Heart Index (VRHi) was introduced as the first quantitative radiographic index specifically designed to assess right-sided cardiac remodelling in dogs. Initial studies demonstrated good reproducibility and diagnostic performance for detecting right heart remodelling, and breed-specific reference intervals have subsequently been established to improve its clinical applicability [18,19]. However, its diagnostic value has not been investigated in dogs with heartworm disease, in which PH-induced right-sided remodelling is a major determinant of disease severity.

Therefore, the aim of this study was to evaluate the diagnostic performance of the VRHi for identifying dogs with a high echocardiographic probability of PH associated with heartworm disease, to assess its association with echocardiographic indices of right-sided cardiac remodelling, and to compare its diagnostic performance with conventional VHS.

## 2. Materials and Methods

### 2.1. Animals

A total of 54 dogs residing on the island of Gran Canaria were included in the study and divided into two groups: a control group, comprising 20 dogs, and a heartworm disease group, comprising 34 dogs. The heartworm disease group was further subdivided equally according to the presence or absence of a high echocardiographic probability of PH (17 dogs in each subgroup).

Identification and clinical data were recorded for each animal, including age, breed, sex, body weight, body surface area (BSA), and cranial/thoracic conformation. In addition, a complete physical examination was performed in all dogs to identify clinical evidence of concomitant disease. The presence or absence of cardiorespiratory signs was recorded, as well as clinical signs compatible with right-sided congestive heart failure (R-CHF), including expiratory dyspnea at rest, ascites, subcutaneous edema, pleural or pericardial effusion, jugular pulse, or caudal vena cava distension. Dogs with clinical or diagnostic imaging findings suggestive of concomitant cardiac or respiratory disorders, or other conditions potentially affecting the cardiovascular system, were excluded from the study.

The study was conducted at the Veterinary Teaching Hospital of the University of Las Palmas de Gran Canaria, located in an area endemic for *D. immitis*, between April 2025 and March 2026 [20]. The inclusion criteria for the study were as follows: (a) age over 7 months, (b) mesocephalic conformation, (c) no administration of medication with cardiorespiratory effects, and (d) absence of other cardiac or respiratory disorders or diseases potentially affecting the cardiovascular system. Additionally, dogs in the heartworm disease group had not received prophylactic treatment against *D. immitis*.

Heartworm infection was confirmed using a commercial immunochromatographic antigen test (Urano Dirofilaria^®^, Urano Vet SL, Barcelona, Spain). Dogs with negative test results were assigned to the control group and were considered cardiovascularly healthy based on physical examination and echocardiographic assessment. Thoracic radiographs in control dogs were obtained as part of the preoperative evaluation for elective surgical procedures unrelated to cardiorespiratory disease, such as neutering or orthopedic procedures.

### 2.2. Radiography

Digital thoracic radiographs were obtained at peak inspiration without sedation or anesthesia to avoid potential alterations in cardiac size and morphology, using a high-frequency X-ray generator (HFQ 300 P, Bennett, NC, USA). Radiographic exposure settings (kVp and mAs) were adjusted individually according to body size, and appropriate radiation protection measures were applied throughout the study.

Four standard thoracic projections were obtained from each dog, including dorsoventral (DV), ventrodorsal (VD), right lateral (RL), and left lateral (LL) views. The RL projection was available in all dogs (*n* = 54), whereas the LL, DV, and VD projections were available in 51, 49, and 46 dogs, respectively. Radiographs showing excessive rotation or motion artifacts at the time of image acquisition were considered unsuitable for reliable VRHi measurement and were excluded from the corresponding projection analysis. In the control group, RL, LL, DV, and VD projections were available in 20, 18, 19, and 16 dogs, respectively, whereas the corresponding numbers in dogs with heartworm disease were 34, 33, 30, and 30.

All radiographic images were exported in DICOM format and analyzed using commercially available software (Horos™ version 4.0.1, Geneva, Switzerland). The VHS was measured in the RL projection according to the method described by Buchanan and Bücheler (1995) [17]. Briefly, the cardiac long and short axes were measured, transposed to the vertebral column beginning at the cranial edge of the fourth thoracic vertebra (T4), and expressed as the sum of vertebral units.

**Figure 1:**
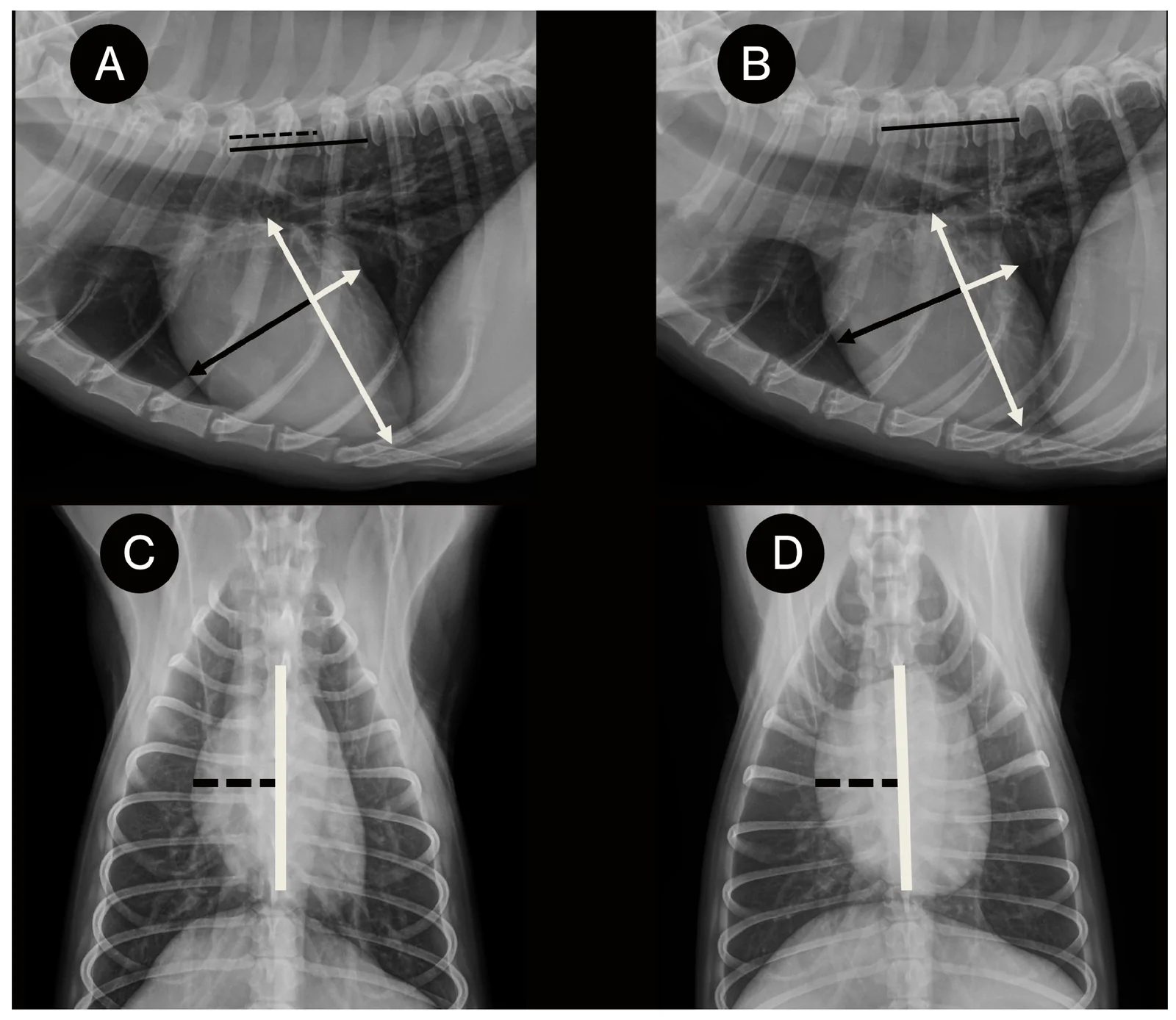
Measurement of the vertebral right heart index (VRHi) on thoracic radiographs of a dog infected with *Dirofilaria immitis*: (**A**) Right lateral (RL) projection. (**B**) Left lateral (LL) projection. The long and maximum short axes of the cardiac silhouette (white arrows) were identified according to the vertebral heart size (VHS) method. In these radiographic views (**A**,**B**), the short-axis distance (black arrow) was traced from the cranial margin of the cardiac silhouette to the long-axis line (white line). Subsequently, the same distance was transposed, starting at the cranial edge of T4 and extending caudally and parallel to the vertebral canal, and its length was expressed in vertebral body units. (**C**) Dorsoventral (DV) projection. (**D**) Ventrodorsal (VD) projection. In the DV and VD projections, the thoracic long axis (white line) was aligned with the vertebral column, and the maximum cardiac short axis was drawn perpendicular to it. The distance from the right cardiac border to the thoracic long axis (black dashed line) was measured and subsequently transposed to the RL projection, beginning at the cranial edge of T4 and extending caudally parallel to the vertebral canal, where it was expressed in vertebral body units to obtain the VRHi.

The Right Vertebral Heart Index (VRHi) was measured in all available radiographic projections following the methodology described by Puccinelli et al. and Choi et al. [18,19] (Figure 1). In the lateral projections (RL and LL), the long and short cardiac axes were identified, and the distance between the cranial border of the cardiac silhouette and the intersection of both axes was measured and transposed to the vertebral column from the cranial edge of T4 to obtain the corresponding vertebral units. In the VD and DV projections, the thoracic long axis was drawn as a line superimposed on the vertebral column. The maximum short axis of the heart was drawn perpendicular to the thoracic long axis, forming a 90° angle between the two axes. Along this short axis, the distance from the right margin of the cardiac silhouette to the intersection with the thoracic long axis was measured and subsequently transposed to the thoracic vertebrae on the RL projection, as previously described [19].

All radiographic measurements were performed by the same investigator (J.I.M.), a professor of Diagnostic Imaging at the University of Las Palmas de Gran Canaria (ULPGC). The investigator was blinded to group allocation, clinical status, and echocardiographic findings at the time of radiographic assessment. Radiographic images were anonymized and evaluated in a randomized order to minimize potential observer bias.

### 2.3. Echocardiography

An echocardiographic examination was performed on all dogs using an ultrasound system equipped with two-dimensional, M-mode, spectral Doppler, and color Doppler imaging, with multifrequency transducers (2.5–12.5 MHz) (Vivid IQ, General Electric, Boston, MA, USA) selected according to body weight. All examinations were performed in conscious dogs, without sedation or anesthesia, in right and left lateral recumbency under continuous electrocardiographic monitoring. At least three consecutive sinus rhythm cardiac cycles were recorded for each measurement. All examinations were performed by the same experienced investigator (J.I.M.). Echocardiographic and thoracic radiographic examinations were performed on the same day in all dogs.

Echocardiography was performed to confirm the presence of adult *D. immitis* worms, estimate relative parasite burden, exclude concurrent cardiovascular diseases, assess the probability of PH, and evaluate right-sided cardiac remodelling.

Relative parasite burden was graded according to the scoring system described by Venco et al. [21], using a four-point scale: grade 1, no worms visualized; grade 2, few compatible worm images in the distal right pulmonary artery; grade 3, worm images extending from the right pulmonary artery to the main pulmonary artery; and grade 4, worms occupying the entire right pulmonary artery and the main pulmonary artery up to the pulmonary valve.

The echocardiographic probability of PH was assessed within the framework proposed by the ACVIM consensus statement [4]. The right pulmonary artery distensibility index (RPADi) and tricuspid regurgitation pressure gradient (TRPG) were measured using previously described techniques [8,22]. For the purposes of the present study, an operational definition was established whereby dogs were classified as having a high echocardiographic probability of PH only when both TRPG > 46 mmHg (TRV > 3.4 m/s) and RPADi < 30% were present. RPADi < 30% was used as an additional echocardiographic sign of PH at the level of the pulmonary artery, in accordance with the ACVIM consensus framework [4]. Based on these criteria, dogs in the heartworm disease group were classified according to the presence or absence of a high echocardiographic probability of PH.

Furthermore, echocardiographic measurements were obtained to assess right-sided cardiac remodelling. The right ventricular end-diastolic area (RVEDA), right ventricular free wall thickness in diastole (RVWTd), and right atrial area (RAAi) were measured using previously described techniques and normalized for body weight as RVEDAi (cm^2^/kg^0.665^), RVWTdi (cm/kg^0.250^), and RAAi (cm^2^/kg^0.71^), according to published reference methods [23].

### 2.4. Ethical Statement

Written informed consent was obtained from all owners before their dogs were included in the study. This observational study was based exclusively on diagnostic procedures performed as part of routine veterinary clinical practice, and no additional interventions or procedures were carried out for research purposes. In control dogs, thoracic radiographs were obtained as part of their clinical/preoperative protocol independently of their subsequent inclusion in the study, whereas in heartworm-infected dogs, radiographs were obtained as part of the routine diagnostic evaluation of the disease. Therefore, approval by an institutional Animal Ethics Committee was not required. All procedures complied with the applicable Spanish and European legislation on animal welfare.

### 2.5. Statistical Analysis

Statistical analysis was performed using IBM SPSS Statistics software (version 25.0; IBM Corp., Armonk, NY, USA). Categorical variables, including sex, presence of clinical signs, presence of R-CHF, parasite burden, and other dichotomous variables derived from echocardiographic criteria, were described using absolute frequencies and percentages. Comparisons of categorical variables among groups were performed using the chi-square test. Continuous variables were summarized using mean, standard deviation, median, interquartile range, minimum, and maximum values. The normality of quantitative variables was assessed using the Shapiro–Wilk test.

As most variables did not follow a normal distribution, comparisons among the three independent groups were performed using the Kruskal–Wallis test, followed by Dunn’s post hoc test with adjustment for multiple comparisons. These tests were used to compare radiographic parameters (VHS, VRHi RL, VRHi LL, VRHi DV, and VRHi VD) and echocardiographic parameters (RPADi, RAAi, RVEDAi, and RVWTdi) among healthy dogs and dogs infected with *D. immitis*, with or without a high echocardiographic probability of PH.

Associations between quantitative variables were assessed by correlation analysis. Both Pearson’s and Spearman’s correlation coefficients were calculated according to the nature and distribution of the variables analyzed. The strength and direction of the correlations were determined using the corresponding correlation coefficients: r for Pearson’s correlation and ρ for Spearman’s correlation.

Receiver operating characteristic (ROC) curve analysis was performed to evaluate the diagnostic ability of VHS in the RL projection and VRHi in the different radiographic projections to identify dogs with a high echocardiographic probability of PH. The area under the curve (AUC) was calculated for each projection, and optimal cut-off values were determined using Youden’s index, with the corresponding sensitivity and specificity values obtained. The AUCs of VRHi-RL and VHS-RL were compared using DeLong’s test for correlated ROC curves.

For all analyses, statistical significance was set at *p* < 0.05.

## 3. Results

A total of 54 dogs were included in the study, of which 25 (46.3%) were females and 29 (53.7%) were males. Ages ranged from 1 to 13 years, with a mean age of 5.67 years. Dogs of different breeds were included, all of which were mesocephalic, with mixed-breed dogs being the most frequent breed type (30/54; 55.6%). Body weight ranged from 2.9 to 40 kg, with a mean body weight of 17.35 kg.

When comparing the three study groups (controls, dogs with heartworm disease without a high echocardiographic probability of PH, and dogs with heartworm disease with a high echocardiographic probability of PH), no significant differences were observed in sex, age, body weight, or body surface area (BSA), indicating a homogeneous distribution of the main demographic variables among the groups. In contrast, clinical signs were observed in 23.5% of dogs with heartworm disease without a high echocardiographic probability of PH and in 88.2% of dogs with a high echocardiographic probability of PH (*p* < 0.001). Similarly, R-CHF was observed only in dogs with a high echocardiographic probability of PH, affecting 41.2% of animals in this group (*p* < 0.001). Regarding parasite burden, dogs with a high echocardiographic probability of PH tended to have a higher parasite burden, although the difference between heartworm-infected groups was not statistically significant (*p* = 0.079) (Table 1).

Echocardiographic parameters showed significant differences among the three study groups (Table 2). Dogs infected with *D. immitis* with a high echocardiographic probability of PH had significantly higher RAAi, RVEDAi, and RVWTdi values than both healthy dogs and infected dogs without a high echocardiographic probability of PH (*p* < 0.001 for all comparisons). These dogs also had significantly lower RPADi and higher TRPG values (*p* < 0.001), consistent with the echocardiographic criteria used for their classification.

Right-sided cardiac remodelling parameters differed among the three groups, with dogs with heartworm disease and PH showing the highest RAAi, RVEDAi, and RVWTdi values.

Radiographic parameters also showed significant differences among groups (Table 3). The VHS in the right lateral projection was significantly higher in dogs infected with *D. immitis* with a high echocardiographic probability of PH compared with both healthy dogs and infected dogs without PH (*p* = 0.002). The VRHi values differed significantly among the three groups in all evaluated projections (RL, LL, DV, and VD), with the highest values observed in dogs with heartworm disease and a high echocardiographic probability of PH (*p* < 0.05 for all overall comparisons).

The TRPG showed strong positive correlations with RAAi (rs = 0.820; *p* < 0.001), RVEDAi (rs = 0.826; *p* < 0.001), and RVWTdi (rs = 0.814; *p* < 0.001). In contrast, RPADi showed strong negative correlations with RAAi (rs = −0.826; *p* < 0.001), RVEDAi (rs = −0.818; *p* < 0.001), and RVWTdi (rs = −0.819; *p* < 0.001). Overall, higher TRPG values and lower RPADi values were associated with increased right atrial size, right ventricular dilation, and right ventricular free wall thickness.

Significant positive correlations were observed between VRHi measurements and echocardiographic parameters of right-sided cardiac remodelling. Overall, the correlation coefficients ranged from moderate to strong across the different radiographic projections. The VD projection showed the strongest associations with the three echocardiographic parameters evaluated: RAAi (rs = 0.653; *p* < 0.001), RVEDAi (rs = 0.653; *p* < 0.001), and RVWTdi (rs = 0.656; *p* < 0.001). Similarly, VRHi measured in the RL projection showed statistically significant correlations with RAAi (rs = 0.600), RVEDAi (rs = 0.593), and RVWTdi (rs = 0.588) (*p* < 0.001). Overall, higher VRHi values were associated with higher RAAi, RVEDAi, and RVWTdi values in all radiographic projections analyzed.

**Figure 2:**
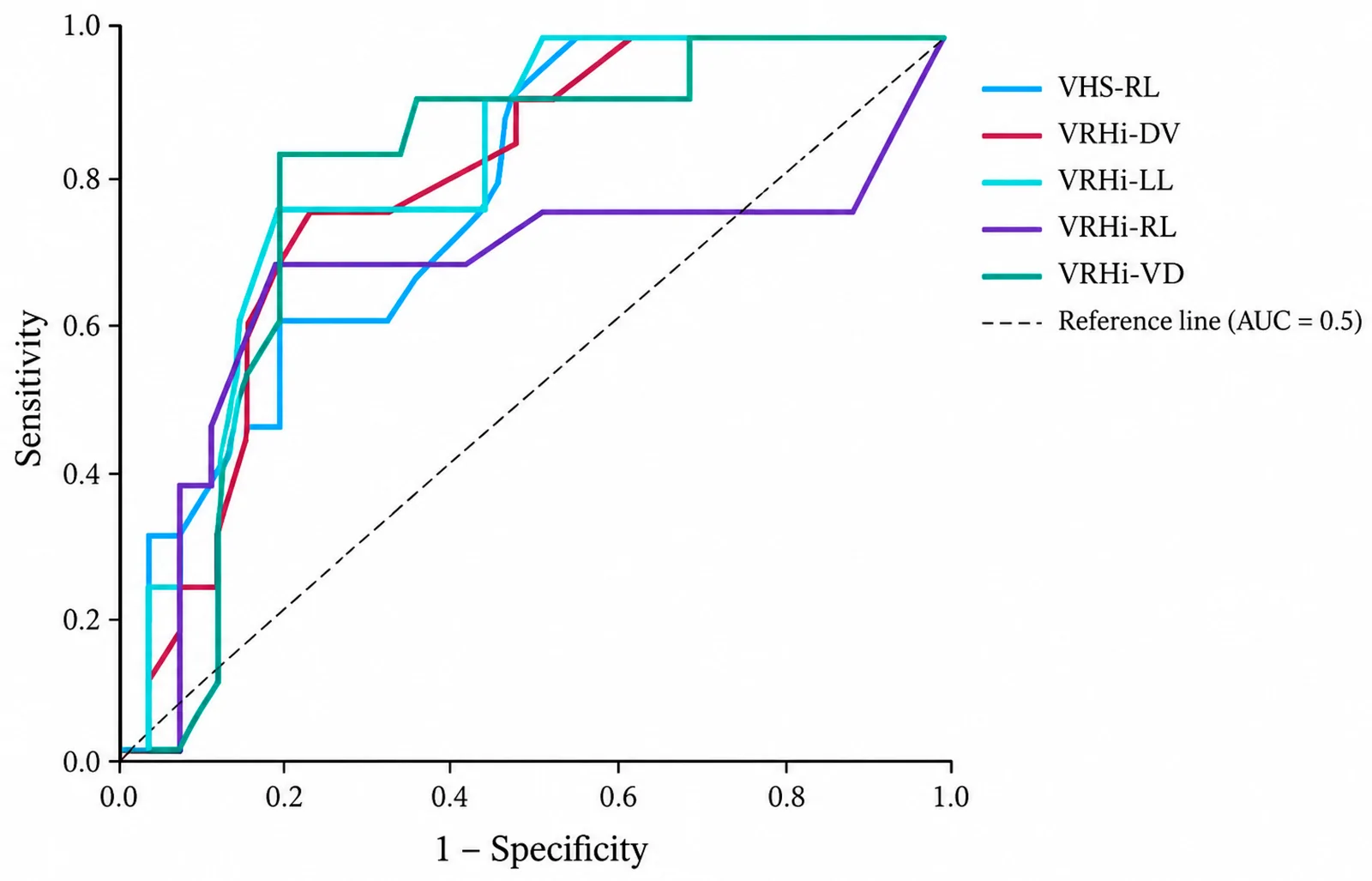
Receiver operating characteristic curves for VHS and VRHi for the identification of high echocardiographic probability of pulmonary hypertension. Receiver operating characteristic (ROC) curves for the vertebral heart size (VHS) in the right lateral (RL) projection and the Vertebral Right Heart Index (VRHi) in the right lateral (RL), left lateral (LL), dorsoventral (DV), and ventrodorsal (VD) projections. Sensitivity is plotted against 1 − specificity. The diagonal dashed line represents the reference line corresponding to an AUC of 0.5.

The ROC curve analysis showed that all radiographic indices evaluated had significant discriminatory ability to identify dogs with a high echocardiographic probability of PH, as defined by the operational echocardiographic criteria used in this study (TRPG > 46 mmHg and RPADi < 30%) (Table 4) (Figure 2). The VRHi measured in the RL projection showed an AUC of 0.835 (95% CI: 0.716–0.933; *p* < 0.001), which was numerically higher than that obtained for the other evaluated radiographic indices. The optimal cut-off value was ≥3.3 vertebral bodies, with a sensitivity of 76.9% and a specificity of 82.1%. The VD projection also showed good discriminatory ability (AUC = 0.813; 95% CI: 0.663–0.938; *p* < 0.001), with a sensitivity of 69.6% and a specificity of 91.3% at a cut-off value of ≥2.8 vertebral bodies. The LL and DV projections also showed discriminatory ability for identifying dogs with a high echocardiographic probability of PH, although their AUC values were numerically lower than those obtained for VRHi in the RL and VD projections. Conventional VHS showed an AUC of 0.764 (95% CI: 0.628–0.877; *p* < 0.001), with high sensitivity (96.2%) but lower specificity (50.0%). Although the AUC of VRHi-RL was numerically higher than that of VHS, DeLong’s test showed no statistically significant difference between the two AUCs (*p* = 0.519).

## 4. Discussion

The present study evaluated the diagnostic value of VRHi for assessing right-sided cardiac remodelling associated with PH in dogs with heartworm disease. The main findings were that VRHi differed among the study groups in all evaluated radiographic projections and was positively correlated with echocardiographic indices of right-sided cardiac remodelling. In addition, dogs with heartworm disease and a high echocardiographic probability of PH showed more marked right-sided cardiac remodelling and a higher frequency of clinical signs than infected dogs without PH and healthy dogs. ROC analysis further supported the diagnostic usefulness of VRHi for identifying dogs with a high echocardiographic probability of PH, particularly in the RL and VD projections.

Likewise, the positive correlations observed between TRPG and the indices of right-sided cardiac remodelling, together with the negative correlation between these indices and RPADi, support the close relationship between echocardiographic indicators of PH and more marked right-sided cardiac remodelling, in accordance with the ACVIM consensus recommendations and previous studies [4,23,24].

Although dogs with a high echocardiographic probability of PH tended to show a higher parasite burden than dogs without a high echocardiographic probability of PH, this difference was not statistically significant (*p* = 0.079). Therefore, this finding should be interpreted cautiously, particularly given the small number of dogs in some parasite-burden categories. Moreover, the severity of heartworm disease also depends on factors such as the duration of infection, the host inflammatory response, parasite location, and the degree of chronic vascular injury [1,8,25].

In this context, VHS is the most widely used objective radiographic measurement for estimating cardiac silhouette size [17]. In our study, dogs with a high echocardiographic probability of PH showed higher VHS values, consistent with right-sided cardiac remodelling associated with pressure overload. However, since VHS assesses overall cardiac size, its ability to specifically detect right heart alterations is limited. Moreover, factors such as breed, thoracic conformation, and radiographic projection may influence radiographic cardiac measurements, including both VHS and VRHi [19,26,27].

One of the main findings of the present study was that VRHi values were significantly higher in dogs with heartworm disease and a high echocardiographic probability of PH. This index, recently proposed as the first quantitative radiographic method specifically designed to assess right heart enlargement in dogs [18], showed values in our population that were comparable to those previously described. Some differences in cut-off values and diagnostic accuracy were observed between our results and those reported by Puccinelli et al. [18]. These differences may be related to the characteristics of the study populations. Puccinelli et al. [18] included dogs with right heart remodelling associated with different underlying diseases, whereas the present study included only dogs with cardiopulmonary dirofilariosis. In these dogs, right-sided cardiac remodelling is mainly associated with precapillary PH secondary to pulmonary vascular lesions induced by *D. immitis*. As breed and thoracic conformation may influence VRHi values [19], only mesocephalic dogs were included in the present study to minimize this potential source of variability. However, this selection limits the generalizability of our findings to dogs with other thoracic conformations.

Moreover, the VRHi was positively correlated with the main echocardiographic indices of right-sided cardiac remodelling (RAAi, RVEDAi, and RVWTdi), supporting an association between radiographic right heart size and structural alterations detected by echocardiography.

The ROC curve analysis supported the diagnostic usefulness of VRHi for identifying dogs with a high echocardiographic probability of PH among dogs with heartworm disease. The RL projection showed a numerically higher AUC than conventional VHS. However, the DeLong comparison showed no statistically significant difference between their AUCs. The VD projection was notable for its high specificity. Nevertheless, because VRHi specifically quantifies the right-sided component of the cardiac silhouette, it may provide complementary information to VHS, which reflects overall cardiac size. In a disease such as heartworm disease, characterized by the development of PH and progressive right heart remodelling, a measurement specifically focused on these structures may provide complementary radiographic information for the assessment of cardiovascular involvement.

The results obtained are also consistent with previous studies that have linked thoracic radiographic abnormalities with the presence of PH in dogs. Adams et al. [13] described an association between enlargement of the pulmonary arteries and right cardiac chambers and the severity of PH estimated by echocardiography. Similarly, Falcón-Cordón et al. [5] observed that the CrPA/R4 and CdPA/R9 ratios are useful parameters for identifying PH in dogs with heartworm disease. In this context, the present study extends the available evidence by evaluating VRHi as a radiographic index specifically focused on right-sided cardiac remodelling in dogs with cardiopulmonary dirofilariosis.

This study has some limitations that should be considered. First, the sample size was relatively small, particularly after subdividing dogs with heartworm disease according to the presence or absence of a high echocardiographic probability of PH. In addition, only mesocephalic dogs were included, which limits the applicability of the results to brachycephalic or dolichocephalic breeds, in which thoracic conformation may influence radiographic measurements. Another limitation was that not all radiographic projections were available for all dogs, as images showing excessive rotation or motion artifacts were excluded from the analysis. This reduced the sample size for the LL, DV, and VD projections and may have affected the statistical power of the corresponding analyses. Additionally, all radiographs were obtained in conscious, manually restrained dogs; therefore, the applicability of these findings to radiographs obtained under sedation remains to be determined. Intra- and inter-observer variability of VRHi measurements was not assessed in the present study, representing an additional limitation. Although the reproducibility of the VRHi measurement technique has previously been evaluated by Puccinelli et al. [18], reproducibility under the specific imaging and clinical conditions of the present study remains to be established. No a priori sample size calculation was performed; therefore, the present study should be considered exploratory, and the diagnostic performance estimates, particularly for projections with fewer available radiographs, should be interpreted with caution. All radiographic measurements were performed by a single experienced observer who was blinded to group allocation, clinical status, and echocardiographic findings, with images evaluated in randomized order to minimize potential observer bias. Radiographic measurements may be influenced by image quality, degree of inspiration, and animal positioning. Although radiographs were obtained as close as possible to peak inspiration, no specific equipment was available to control the respiratory phase; therefore, this variable could not be fully standardized and may have influenced some results.

Future studies should include a larger number of animals and different thoracic conformations, obtain all radiographic projections, and, if possible, implement stricter control of the respiratory phase during image acquisition. Longitudinal studies evaluating VRHi before and after adulticide treatment would also be valuable to determine whether this index could be used not only as a diagnostic tool but also as a follow-up parameter for monitoring changes in right-sided cardiac remodelling.

Overall, the results of the present study suggest that VRHi, particularly in the RL and VD projections, may be useful for identifying dogs with heartworm disease and a high echocardiographic probability of PH. Its association with echocardiographic parameters of right-sided cardiac remodelling and its diagnostic performance support its potential role as an objective complementary radiographic measurement in these patients. However, VRHi should not be considered a substitute for echocardiography, which remains essential for the comprehensive assessment of suspected PH.

## 5. Conclusions

The present study suggests that VRHi could be a useful radiographic parameter to aid in the identification of dogs with a high echocardiographic probability of PH associated with heartworm disease. VRHi measurements were significantly associated with echocardiographic indices of right-sided cardiac remodelling, while the RL and VD projections showed the highest observed AUC values among the VRHi measurements evaluated. These findings support the potential use of VRHi as an objective complementary radiographic measurement in dogs with heartworm disease and suspected PH, while echocardiography remains necessary for a comprehensive assessment of PH probability and right-sided cardiac remodelling.

## Figures and Tables

**Table 1:** Clinical characteristics of the study population according to study group.

Variable	Category	Control (*n* = 20)	Heartworm Disease Without PH (*n* = 17)	Heartworm Disease with PH (*n* = 17)	*p*-Value
Sex	Males	11 (55%)	8 (47.1%)	10 (58.8%)	0.781
	Females	9 (45%)	9 (52.9%)	7 (41.2%)	
Symptomatic dogs		0 (0%)	4 (23.5%)	15 (88.2%)	<0.001
R-CHF		0 (0%)	0 (0%)	7 (41.2%)	<0.001
Parasite burden	Grade 1	NA	1 (5.9%)	0 (0%)	0.079
	Grade 2	NA	12 (70.6%)	8 (47.1%)	
	Grade 3	NA	4 (23.5%)	4 (23.5%)	
	Grade 4	NA	0 (0%)	5 (29.4%)	
Age		5.5 (4–8) years	5 (2–10) years	4 (3–8) years	0.663
Body weight		17.7 (14.3–23.5) kg	11.5 (9.4–26.0) kg	12.0 (7.5–21.4) kg	0.208
BSA		0.67 (0.58–0.81) m^2^	0.51 (0.44–0.87) m^2^	0.52 (0.38–0.76) m^2^	0.208

Legend: Categorical variables are presented as numbers of dogs (percentages), whereas continuous variables are presented as medians (25th–75th percentiles). The letter *n* indicates the total number of dogs included in each study group. Parasite burden was compared only between heartworm-infected groups. Abbreviations: BSA, body surface area; PH, pulmonary hypertension; NA, not applicable; R-CHF, right-sided congestive heart failure.

**Table 2:** Comparison of echocardiographic parameters of right-sided cardiac remodelling among the study groups.

Variable	Control (*n* = 20) Median (25th–75th Percentile)	Heartworm Disease Without PH (*n* = 17) Median (25th–75th Percentile)	Heartworm Disease with PH (*n* = 17) Median (25th–75th Percentile)	Overall *p*-Value
RAAi	0.60 (0.54–0.68) ^a^	0.74 (0.59–0.82) ^a^	1.42 (1.30–1.60) ^b^	<0.001 *
RVEDAi	1.09 (1.00–1.20) ^a^	1.32 (1.05–1.43) ^a^	2.15 (2.05–2.35) ^b^	<0.001 *
RVWTdi	0.31 (0.29–0.36) ^a^	0.38 (0.30–0.41) ^a^	0.55 (0.52–0.60) ^b^	<0.001 *
RPADi	41.19 (37.47–45.15)% ^a^	28.00 (25.00–37.00)% ^b^	15.00 (11.10–20.00)% ^c^	<0.001 *
TRPG	3.36 (1.14–5.15) mmHg ^a^	8.18 (0–22.28) mmHg ^a^	66.00 (56.97–74.50) mmHg ^b^	<0.001 *

Legend: Continuous variables are presented as medians (25th–75th percentiles). The letter *n* indicates the total number of dogs included in each study group. Different superscript letters (a, b, c) within the same row indicate statistically significant differences between groups in Dunn’s post hoc comparisons with Bonferroni adjustment; groups sharing the same superscript letter are not significantly different. (*) *p* < 0.001. Indexed parameters were normalized to body weight (BW) as follows: RAAi = RAA/BW^0.71^; RVEDAi = RVEDA/BW^0.665^; RVWTdi = RVWTd/BW^0.25^. Abbreviations: BW, body weight; PH, pulmonary hypertension; RAA, right atrial area; RAAi, right atrial area index; RPADi, right pulmonary artery distensibility index; RVEDA, right ventricular end-diastolic area; RVEDAi, right ventricular end-diastolic area index; RVWTd, right ventricular wall thickness in diastole; RVWTdi, right ventricular wall thickness in diastole index; TRPG, tricuspid regurgitation pressure gradient.

**Table 3:** Comparison of radiographic parameters among the study groups.

Variable	Projection	Control (*n* = 20) Median (25th–75th Percentile)	Heartworm Disease Without PH (*n* = 17) Median (25th–75th Percentile)	Heartworm Disease with PH (*n* = 17) Median (25th–75th Percentile)	*p*-Value
VHS (v)	RL	9.8 (9.3–10.2) ^a^	10.0 (9.7–10.8) ^a^	11.0 (10.0–11.2) ^b^	0.002
VRHi (v)	RL	3.10 (2.90–3.20) ^a^	3.30 (3.10–3.50) ^b^	3.80 (3.50–4.00) ^c^	<0.001 *
	LL	3.15 (3.00–3.20) ^a^	3.40 (3.20–3.60) ^b^	3.80 (3.50–4.00) ^c^	0.017
	DV	2.20 (2.10–2.40) ^a^	2.50 (2.20–2.80) ^b^	3.00 (2.80–3.30) ^c^	<0.001 *
	VD	2.30 (2.10–2.50) ^a^	2.60 (2.30–2.90) ^b^	3.10 (2.90–3.40) ^c^	<0.001 *

Legend: Values with different superscript letters (a, b, c) within the same row indicate significant differences between groups after post hoc analysis. *p* < 0.05; (*) *p* < 0.001. Abbreviations: v, vertebral bodies; DV, dorsoventral projection; RL, right lateral projection; LL, left lateral projection; VD, ventrodorsal projection; VHS, vertebral heart size; VRHi, Vertebral Right Heart Index.

**Table 4:** Diagnostic ability of radiographic parameters to detect overall high echocardiographic probability of pulmonary hypertension. Data were obtained by ROC curve analysis.

Variable	Projection (*n*)	AUC	95% CI	Optimal Cut-Off	Sensitivity, % (95% CI)	Specificity, % (95% CI)	*p*-Value
VHS	RL (54)	0.764	0.628–0.877	≥9.8	96.2 (80.4–99.9)	50.0 (30.6–69.4)	<0.001 *
VRHi	RL (54)	0.835	0.716–0.933	≥3.3	76.9 (56.4–91.0)	82.1 (63.1–93.9)	<0.001 *
	LL (51)	0.742	0.592–0.880	≥3.5	60.0 (38.7–78.9)	92.3 (74.9–99.1)	0.003
	DV (49)	0.706	0.540–0.854	≥2.7	59.1 (36.4–79.3)	81.5 (61.9–93.7)	0.014
	VD (46)	0.813	0.663–0.938	≥2.8	69.6 (47.1–86.8)	91.3 (72.0–98.9)	<0.001 *

Legend: For the RL projection (*n* = 54), the ROC analysis included 17 positive cases (heartworm-infected dogs with a high echocardiographic probability of PH) and 37 negative cases (20 healthy controls and 17 heartworm-infected dogs without a high echocardiographic probability of PH). *p* < 0.05; (*) *p* < 0.001. Abbreviations: AUC, area under the curve; CI, confidence interval; v, vertebral bodies; DV, dorsoventral projection; LL, left laterolateral projection; N, number; RL, right laterolateral projection; VD, ventrodorsal projection; VHS, vertebral heart size; VRHi, Vertebral Right Heart Index.

## Data Availability

The data presented in this study are available within the article. Additional data are available from the corresponding author upon reasonable request.
